# Amphibians and reptiles of the state of Durango, Mexico, with comparisons with adjoining states

**DOI:** 10.3897/zookeys.748.22768

**Published:** 2018-04-04

**Authors:** Julio A. Lemos-Espinal, Geoffrey R. Smith, Hector Gadsden-Esparza, Rosaura Valdez-Lares, Guillermo A. Woolrich-Piña

**Affiliations:** 1 Laboratorio de Ecología-UBIPRO, FES Iztacala UNAM, Avenida los Barrios 1, Los Reyes Iztacala, Tlalnepantla, edo. de México, Mexico – 54090; 2 Department of Biology, Denison University, Granville, OH, USA; 3 Instituto de Ecología, A.C.-Centro Regional del Bajío, Av. Lázaro Cárdenas No 253 A.P. 386 C.P. 61600 Pátzcuaro, Michoacán, Mexico; 4 Centro Interdisciplinario de Investigación para el Desarrollo Integral Regional (CIIDIR), Instituto Politécnico Nacional, Unidad Durango. Sigma 119, Fracc. 20 de Noviembre II, C.P. 34220. Durango, Durango, Mexico; 5 Laboratorio de Zoología. División de Biología. Subdirección de Investigación y Posgrado. Instituto Tecnológico Superior de Zacapoaxtla. Carretera Acuaco Zacapoaxtla Km. 8, Col. Totoltepec, Zacapoaxtla, Puebla 73680, México

**Keywords:** Checklist, Chihuahuan Desert, conservation status, herpetofauna, shared species, Sierra Madre Occidental

## Abstract

A summary of the species of amphibians and reptiles of Durango, as well as their geographic distributions, habitat, and conservation status have been compiled. The herpetofauna of Durango consists of 36 species of amphibians and 120 species of reptiles. Durango shares the most species with Chihuahua (74.0%), and shares fewer species with Sinaloa (48.0%), Nayarit (48.7%), and Coahuila (48.0%). Arid-semiarid and Sierras habitat types have the most species, with valleys and Quebradas habitat types having fewer species. In Durango, there are several taxa of particular conservation concern including eleutherodactylid frogs, eublepharid, iguanid, phrynosomatid, and xantusid lizards, boid, colubrid, and natricid snakes, and emydid and testudinid turtles.

## Introduction

**Figure 1.**
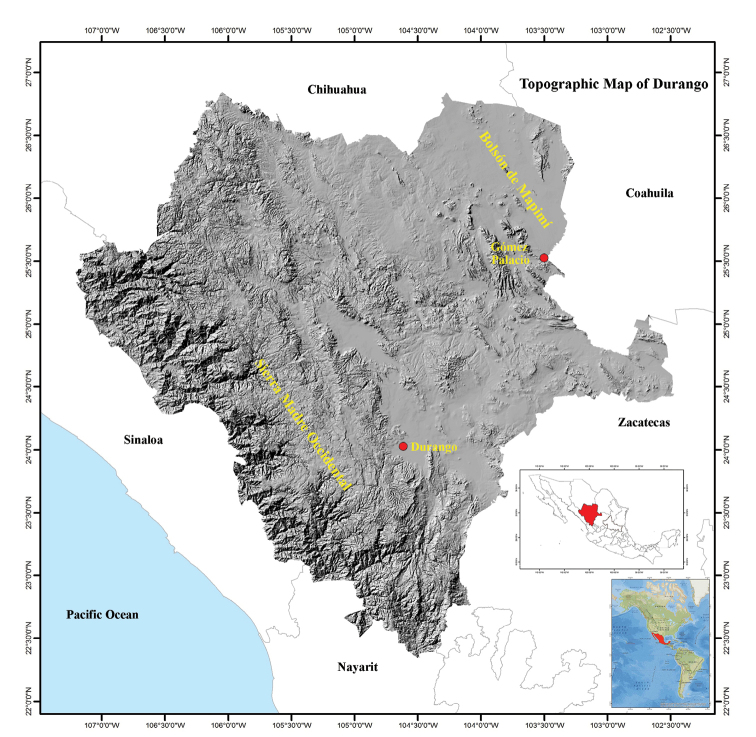
Topographical map of the state of Durango, Mexico (INEGI 2001). Map of America modified from http://www.gifex.com/fullsize/2009-09-17-3/Mapa-de-Amrica.html; Map of Mexico with the state of Durango in red modified from Comisión Nacional para el Conocimiento y Uso de la Biodiversidad (2008).

**Figure 2.**
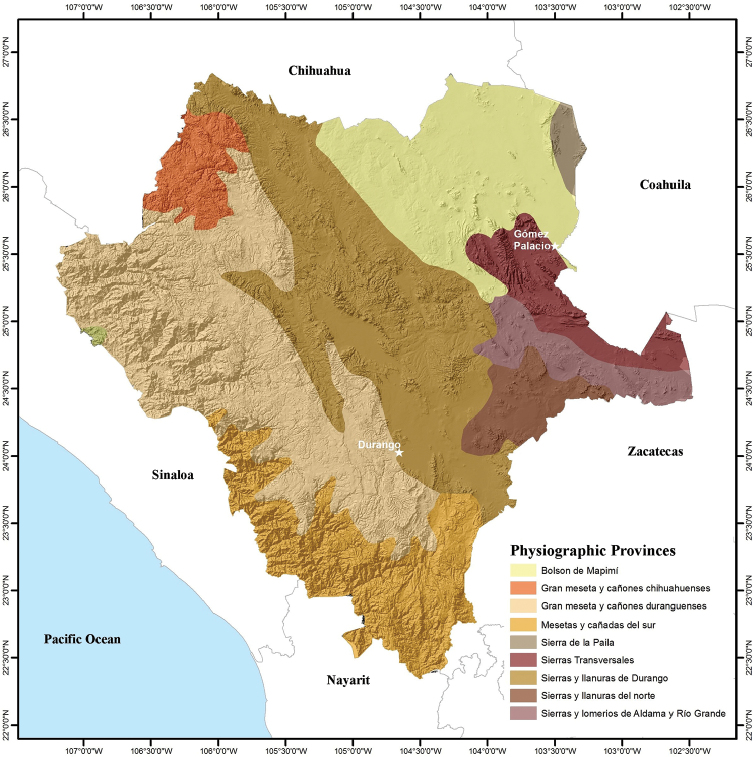
Physiographic provinces of the state of Durango, Mexico (modified from Cervantes-Zamora et al. 1990).

**Figure 3.**
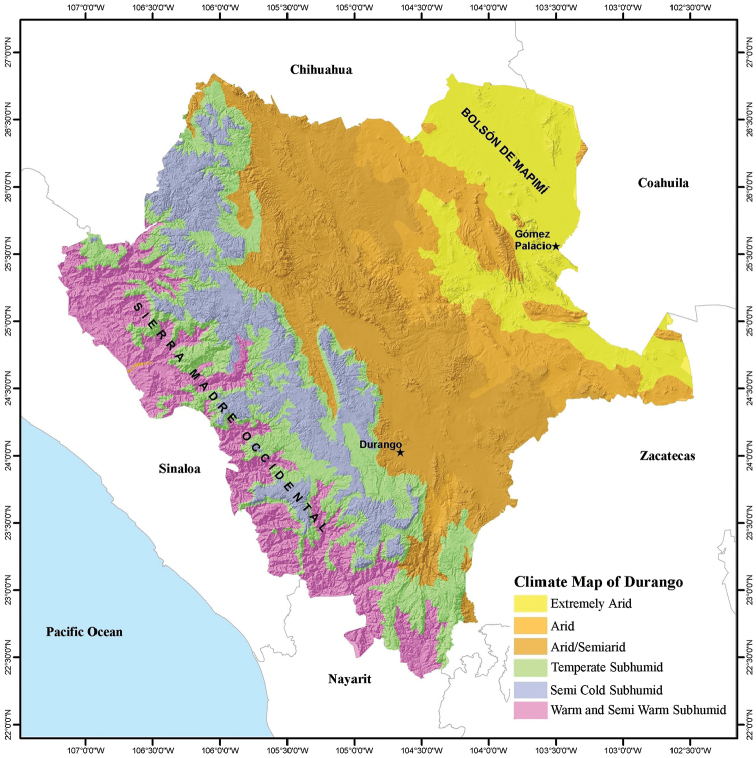
Climate map of the state of Durango, Mexico (modified from García – Comisión Nacional para el Conocimiento y Uso de la Biodiversidad 1998).

**Figure 4.**
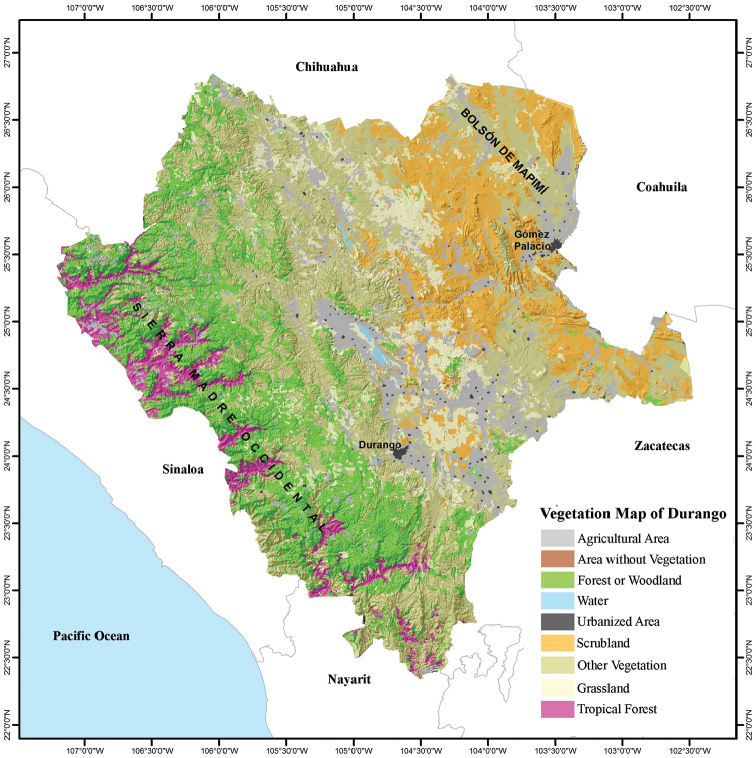
Vegetation map of the state of Durango, Mexico (modified from Dirección General de Geografía – INEGI 2005).

Durango is located in central-northwestern Mexico, and covers 123,317 km^2^ between 22°20'42"N, 26°50'42"N, and 102°28'22"W and 107°12'36"W (Fig. 1). It is the 4^th^ largest state in Mexico, representing 6.3% of the country’s territory. Durango is bordered by Chihuahua to the north, Coahuila to the northeast, Zacatecas to the southeast, Nayarit to the southwest, and Sinaloa to the west (Figs 2–4). Durango has great biodiversity, a consequence of the combination of its geographical location and complex topography. The Tropic of Cancer passes through the southern part of the state, and the Sierra Madre Occidental runs from north to south dividing Durango into three large climatic regions (warm, temperate, and arid-semiarid). Winds from the Pacific Ocean interact with the Sierra Madre Occidental, producing a rain shadow that results in a significant humidity gradient in the state. This gradient results in a great contrast in the composition of species that inhabit the deep canyons of the western lowlands, the great elevations of the Sierra, the valleys of the foothills of the Sierra, and the arid-semiarid region of the eastern part of the state. The diversity of environmental conditions gives Durango a privileged place in terms of biodiversity. The state is home to dense forests of different timber species, such that, at the national level, Durango is the main producer of wood, contributing 28.5% of the total lumber production of the country (INEGI 2016). The Sierra Madre Occidental is considered a center of biodiversity in the North American continent, mainly due to its floral richness (Felger and Wilson 1994).

Topographically, Durango can be divided into four zones arranged (Fig. 1). In the westernmost zone adjacent to Sinaloa and Nayarit, ravines and canyons have formed through millions of years erosion by the rivers that run from the Sierra Madre Occidental to the Pacific Ocean. The southern part of this region is known as the Quebradas. To the east of the Quebradas is the Sierra region containing the mountainous massif of the Sierra Madre Occidental, running from the northwestern corner to the southern tip of Durango. The eastern foothills of the Sierra Madre Occidental are part of the Valley region. In the northeastern quarter of Durango is the arid-semiarid region, which includes the Bolsón de Mapimí. The Bolsón de Mapimí is a region that hosts a number of unique endemic species of lizards and turtles, such as *Uma paraphygas* (Chihuahua Fringe-toed Lizard), *Kinosternon durangoense* (Durango Mud Turtle), and *Gopherus flavomarginatus* (Bolson Tortoise), among other species. South of this region, the physiographic province of the Sierra Madre Oriental enters the eastern part of the state. It is in Durango that the Sierra Madre Occidental and Oriental come closest in Mexico, the western most branch of the Sierra Madre Oriental in eastern Durango is also home of a unique assemblage of lizard species such as *Sceloporus maculosus* (Northern Snub-nosed Spiny Lizard), *Xantusia bolsonae* (Bolson Night Lizard), and *X. extorris* (Durango Night Lizard) (Lemos-Espinal et al. 2017; Valdez-Lares et al. 2013).

These characteristics of the state of Durango have contributed to the presence of a relatively high diversity of amphibian and reptile species, three of which are endemic to the state (*Xantusia bolsonae* [Bolson Night Lizard], *Adelophis foxi* [Fox’s Mountain Meadow Snake], and *Thamnophis nigronuchalis* [Southern Durango Spotted Gartersnake]), or are limited to a small region including Durango and part of one or more of the adjacent states (*Incilius mccoyi* [McCoy’s Toad], *Craugastor tarahumaraensis* [Tarahumara Barking Frog], *Eleutherodactylus pallidus* [Pale Chirping Frog], *E. saxatilis* [Marbled Peeping Frog], *Sceloporus lemosespinali* [Lemos-Espinal’s Spiny Lizard], *S. maculosus* [Spotted Spiny Lizard], *S. shannonorum* [Shannons’ Spiny Lizard], *Uma paraphygas* [Chihuahuan Fringe-toed Lizard], *Xantusia extorris* [Durango Night Lizard], *Lampropeltis webbi* [Webb’s Kingsnake], *Thamnophis errans* [Mexican Wandering Gartersnake], *T. unilabialis* [Madrean Narrow-headed Gartersnake], *Crotalus stejnegeri* [Sinaloan Long-tailed Rattlesnake], *Kinosternon durangoense* [Durango Mud Turtle], and *Gopherus flavomarginatus* [Bolson Tortoise]).

Here, the list of amphibians and reptiles that have been recorded in the state of Durango to date is reported upon. While checklists of the herpetofauna of Durango are available (e.g., Valdez-Lares et al. 2013, 2017a, b), these earlier efforts are expanded upon by collecting and by summarizing the conservation statuses and their distributions within the state as well as the global distribution for each documented species. The herpetofauna of Durango is compared to those of the four adjoining states for which recent checklists are available (Chihuahua, Sinaloa, Nayarit, and Coahuila). Our goal is to place this checklist into a regional and conservation context not available in previous publications.

## Materials and methods

A list of amphibians and reptiles of the state of Durango was compiled from the following sources: (1) our own field work; (2) specimens from the Herpetological Collection of CIIDIR-IPN-Durango; (3) databases from the Comisión Nacional para el Conocimiento y Uso de la Biodiversidad (National Commission for the Understanding and Use of Biodiversity; CONABIO), including records from the following 22 collections Colección Herpetológica, Departamento de Zoología, Escuela Nacional de Ciencias Biológicas (ENCB); Colección Herpetológica, Zoological Institute of the Russian Academy of Sciences, St Petersburg, Russia, Facultad de Ciencias UNAM (MZFC-UNAM); Colección Nacional de Anfibios y Reptiles, Instituto de Biología UNAM (CNAR); Amphibians and Reptiles Collection, University of Arizona (UAZ); Collection of Herpetology, Amphibians and Reptiles Section, Carnegie Museum of Natural History, Pittsburgh; Collection of Herpetology, Biology Department, Tulane University, New Orleans (TU); Collection of Herpetology, Department of Vertebrate Zoology, National Museum of Natural History, Smithsonian Institution (USNM); Collection of Herpetology, Herpetology Department, American Museum of Natural History (AMNH); Collection of Herpetology, Herpetology Department, California Academy of Sciences (CAS); Collection of Herpetology, Museum of Comparative Zoology, Harvard University Cambridge (MCZ); Collection of Herpetology, Museum of Vertebrate Zoology, Division of Biological Sciences, University of California Berkeley (MVZ); Collection of Herpetology, Museum of Zoology, University of Michigan Ann Arbor (UMMZ); Collection of Herpetology, Texas Cooperative Wildlife Collection, Texas A&M University (TCWC); Collection of Herpetology, Texas Natural History Collection, University of Texas Austin (TNHC); Collection of Herpetology, University of Colorado Museum (UCM); Collection of Herpetology, University of Illinois Museum of Natural History (UIMNH); Division of Amphibians and Reptiles, Field Museum of Natural History (FMNH); Fort Worth Museum of Sciences and History (FWMSH); Herpetology Section, Natural History Museum of Los Angeles County (LACM); Louisiana State University, Museum of Life Sciences; Merriam Museum, University of Texas Arlington (UTAMM); Museum of Natural History, Division of Herpetology, Kansas University (MNHUK); and (4) a thorough examination of the available literature on amphibians and reptiles in the state.

Species were included in the checklist only if the record was confirmed, either by direct observation or through documented museum records or vouchers in the state. Scientific names used in this publication are based on the taxonomic list published in Lemos-Espinal (2015). The amphibian names follow Frost (2017) or AmphibiaWeb (2017, see paragraphs below) and the reptile names follow Uetz and Hošek (2016). In addition, the conservation status of each species was recorded based on three sources: 1) the IUCN Red List 2017; 2) Environmental Viability Scores from Wilson et al. (2013a,b); 3) listing in SEMARNAT (2010). The following state lists were used to compare the species composition between Durango and the adjoining states: Chihuahua, Lemos-Espinal et al. (2017); Sinaloa, Enderson et al. (2009); Nayarit, Woolrich-Piña et al. (2016); and Coahuila, Lemos-Espinal and Smith (2016). The lists were updated for Chihuahua (adding *P. ornatissimum* (Girard), Montanucci 2015); for Chihuahua and Coahuila (substituting *Sceloporus consobrinus* Baird & Girard for *S. cowlesi* Lowe & Norris, A. Leache, personal communication, April 2017); and for Sinaloa (adding *Gopherus evgoodei*, Edwards et al. 2016). The number of overlapping species between each of these states and Durango was determined, and a cluster analysis used to examine the similarities among the herpetofaunas of Durango and its neighboring states (e.g., Enderson et al. 2009; Smith and Lemos-Espinal 2015).

### Recent taxonomic changes

Acevedo et al. (2016) used two mitochondrial genes and 23 morphometric landmarks to evaluate the taxonomic status of *Rhinella marina*. They demonstrated that there were two separate evolutionary lineages within *R. marina* represented by two distinct morphotypes, one eastern and one western Andean. The concordance between the observed geographic patterns in morphometric and genetic traits support the recognition of two distinct species. The eastern populations retained the name *R. marina*, and the name *R. horribilis* was revalidated for the western populations.

Duellman et al. (2016) treated two major clades as genera (*Hyla*, restricted to the Old World, and *Dryophytes* distributed primarily in the New World but with three species in Asia). *Dryophytes* is therefore used here. In addition, *Sarcohyla bistincta* was originally placed in the genus *Hyla* by Cope (1877), but was moved to the genus *Plectrohyla* by Faivovich et al. (2005). Duellman et al. (2016) performed a phylogenetic analysis of sequences from 503 species of hylid frogs and four outgroup taxa that resulted in a new phylogenetic tree of treefrogs. Among other results, a conservative new classification based on this tree has five new generic names, including *Sarcohyla*. This new genus contains 24 species, most of them from the *Hyla bistincta* and *Hyla arborescandens* groups of Duellman (2001), and includes the new combination *Sarcohyla bistincta*.

The six species of ranid frogs that occur in Durango were long considered to be in the genus *Rana*, however, Frost et al. (2006) recommended the use of the name *Lithobates*, which was controversial. More recently, Yuan et al. (2016) retained all the species of these genera in the traditional genus *Rana*, based on a phylogenetic analysis of six nuclear and three mitochondrial loci sampled from most species of *Rana*, the lack of any diagnostic morphological characters for the genera recognized by Frost et al. (2006), and the clear monophyly of a larger group that include these genera. *Rana* is used here following Yuan et al. (2016) and AmphibiaWeb (2017).

Montanucci (2015) studied the comparative morphology and color pattern variation of short-horned lizards (*Phrynosoma douglasii* species complex) using multivariate analyses of 20 morphological and color-pattern characters, and univariate statistics were summarized for 52 samples. The results of the morphological data analyses supported the recognition of *P. douglasii* as a distinct species, and the resurrection of *P. brevirostris* and *P. ornatissimum* as species distinct from *P. hernandesi*. He recognized two subspecies of *P. ornatissimum*: *P. o. ornatissimum* from central and southern New Mexico and western Texas; and *P. o. brachycercum* from the lower eastern slopes of the Sierra Madre Occidental and the adjacent plains in the Mexican states of Chihuahua, Durango, and Zacatecas.

Tucker et al. (2016), based on Steyskal (1971), explained and justified why the genus name *Aspidoscelis* should be treated as masculine. Names used for species of *Aspidoscelis* occurring in Durango are thus *A. costatus*, *A. gularis*, *A. inornatus*, and *A. marmoratus*.

Card et al. (2016) analyzed the genetic structure and phylogenetic relationships of *Boa* populations using mitochondrial sequences and genome wide SNP data obtained from RADseq, finding evidence that supports three widely-distributed clades roughly corresponding with western North America (Pacific Coast of Mexico), eastern North America (Atlantic Coast of Mexico and Central America), and South America. One of those clades represented the populations of the Pacific slopes of Mexico, from northern Sonora to west of the Isthmus of Tehuantepec. They resurrected the name *sigma* from the population described by Smith (1943) as *Constrictor* (= *Boa*) *constrictorsigma* from the María Madre Island, Tres Marías Islands, Nayarit, Mexico, which was regarded as a junior synonym of *B. c. imperator* by Zweifel (1960). Card et al. (2016) recognized the *Boa* populations from the slopes of the Mexican Pacific as *Boa sigma*, and this is followed here.

## Results and discussion

A total of 156 (three of them introduced) species of amphibians and reptiles is found in Durango. Thirty-six of these species are amphibians (33 anurans [one introduced]), three salamanders) and 120 are reptiles (five turtles, 54 lizards [one introduced], and 61 snakes [one introduced]) (Tables 1, 2). These represent 32 families: eight amphibian (seven anurans; one salamanders), and 23 reptile (12 of lizards [one introduced], eight of snakes [one introduced], and three of turtles). Durango has a total of 73 genera: 14 amphibian (one salamander, 13 anuran), and 59 reptile (22 lizard [one introduced], 34 snake [one introduced], and three turtle). The introduced species are the American Bullfrog (*Rana catesbeiana*), the Mediterranean House Gecko (*Hemidactylus turcicus*), and the Brahminy Blindsnake (*Indotyphlops braminus*).

**Table 1.** Ecoregion (1 = Arid-semiarid; 2 = Valleys; 3 = Sierra; 4 = Quebradas); IUCN Status (DD = Data Deficient; LC = Least Concern, V = Vulnerable, NT = Neat Threatened; E = Endangered; CE = Critically Endangered; NE = not Evaluated) according to the IUCN Red List (The IUCN Red List of Threatened Species, Version 2017-2; www.iucnredlist.org; accessed 9 November 2017), conservation status in Mexico according to SEMARNAT (2010) (P = in danger of extinction, A = threatened; Pr = subject to special protection, NL – not listed), and Environmental Vulnerability Score (EVS – the higher the score the greater the vulnerability: low (L) vulnerability species (EVS of 3–9); medium (M) vulnerability species (EVS of 10–13); and high (H) vulnerability species (EVS of 14–20) from Wilson et al. (2013a,b) and Johnson et al. (2015). Global Distribution: 0 = Endemic to Durango; 1 = Endemic to Mexico; 2 = Shared between the US and Mexico; 3 = widely distributed from Canada or the US to Central or South America; 4 = widely distributed from Mexico to Central America; IN = Introduced.

Taxon	Ecoregion	IUCN	SEMARNAT	EVS	Global
**CLASS AMPHIBIA (36)**					
**ORDER ANURA (33)**					
**Family BUFONIDAE (11)**					
*Anaxyrus cognatus* (Say, 1823)	1,2	LC	NL	L (9)	2
*Anaxyrus compactilis* (Wiegmann, 1833)	2,3	LC	NL	H (14)	1
*Anaxyrus debilis* (Girard, 1854)	1,2	LC	Pr	L (7)	2
*Anaxyrus mexicanus* (Brocchi, 1879)	3	NT	NL	M (13)	1
*Anaxyrus punctatus* (Baird & Girard, 1852)	1,2	LC	NL	L (5)	2
*Anaxyrus woodhousii* (Girard, 1854)	3	LC	NL	M (10)	2
*Incilius marmoreus* (Wiegmann, 1833)	2	LC	NL	M (11)	1
*Incilius mazatlanensis* (Taylor, 1940)	4	LC	NL	M (12)	1
*Incilius mccoyi* Santos-Barrera & Flores-Villela, 2011	2,3	NE	NL	H (14)	1
*Incilius occidentalis* (Camerano, 1879)	2,3,4	LC	NL	M (11)	1
*Rhinella horribilis* (Linnaeus, 1758)	4	NE	NL	L (3)	3
**Family CRAUGASTORIDAE (4)**					
*Craugastor augusti* (Dugès, 1879)	2,4	LC	NL	L (8)	2
*Craugastor occidentalis* (Taylor, 1941)	3	DD	NL	M (13)	1
*Craugastor tarahumaraensis* (Taylor, 1940)	3	V	Pr	H (17)	1
*Craugastor vocalis* (Taylor, 1940)	4	LC	NL	M (13)	1
**Family ELEUTHERODACTYLIDAE (3)**					
*Eleutherodactylus nitidus* (Peters, 1870)	3	LC	NL	M (12)	1
*Eleutherodactylus pallidus* (Duellman, 1958)	4	DD	Pr	H (17)	1
*Eleutherodactylus saxatilis* (Webb, 1962)	3	E	NL	H (17)	1
**Family HYLIDAE (6)**					
*Agalychnis dacnicolor* (Cope, 1864)	4	LC	NL	M (13)	1
*Dryophytes arenicolor* Cope, 1866	2,3,4	LC	NL	L (7)	2
*Dryophytes eximius* (Baird, 1854)	2,3	LC	NL	M (10)	1
*Dryophytes wrightorum* (Taylor, 1938)	3	LC	NL	L (9)	2
*Sarcohyla bistincta* (Cope, 1877)	3	LC	Pr	L (9)	1
*Smilisca baudinii* (Duméril & Bibron, 1841)	4	LC	NL	L (3)	3
**Family MICROHYLIDAE (1)**					
*Gastrophryne olivacea* (Hallowell, 1857)	1	LC	Pr	L (9)	2
**Family RANIDAE (6)**					
*Rana berlandieri* Baird, 1859	1,3	LC	Pr	L (7)	3
*Rana catesbeiana* Shaw, 1802	1	N/A	N/A	N/A	IN
*Rana chiricahuensis* Platz & Mecham, 1979	2,3	V	A	M (11)	2
*Rana magnaocularis* Frost & Bagnara, 1974	4	LC	NL	M (12)	1
*Rana montezumae* Baird, 1854	2	LC	Pr	M (13)	1
*Rana pustulosa* Boulenger, 1833	4	LC	Pr	L (9)	1
**Family SCAPHIOPODIDAE (2)**					
*Scaphiopus couchii* Baird, 1854	1,2	LC	NL	L (3)	2
*Spea multiplicata* (Cope, 1863)	1,2,3	LC	NL	L (6)	2
**ORDER CAUDATA**					
**Family AMBYSTOMATIDAE (3)**					
*Ambystoma rosaceum* Taylor, 1941	3	LC	Pr	H (14)	1
*Ambystoma silvense* Webb, 2004	3	DD	NL	H (14)	1
*Ambystoma velasci* (Dugès, 1888)	2,3	LC	Pr	M (10)	1
**CLASS REPTILIA (120)**					
**ORDER SQUAMATA**					
**SUBORDER LACERTILIA (53)**					
**Family ANGUIDAE (4)**					
*Barisia ciliaris* (Smith, 1942)	2,3	NE	NL	H (15)	1
*Elgaria kingii* Gray, 1838	3	LC	Pr	M (10)	2
*Gerrhonotus infernalis* Baird, 1859	1,3	LC	NL	M (13)	2
*Gerrhonotus liocephalus* Wiegmann, 1828	3	LC	Pr	L (6)	1
**Family CROTAPHYTIDAE (2)**					
*Crotaphytus collaris* (Say, 1823)	1	LC	A	M (13)	2
*Gambelia wislizenii* (Baird & Girard, 1852)	1	LC	Pr	M (13)	2
**Family DACTYLOIDAE (1)**					
*Anolis nebulosus* (Wiegmann, 1834)	3,4	LC	NL	M (13)	1
**Family EUBLEPHARIDAE (2)**					
*Coleonyx brevis* Stejneger, 1893	1	LC	Pr	H (14)	2
*Coleonyx fasciatus* (Boulenger, 1885)	4	LC	NL	H (17)	1
**Family GEKKONIDAE (1)**					
*Hemidactylus turcicus* (Linnaeus, 1758)	1	N/A	N/A	N/A	IN
**Family HELODERMATIDAE (1)**					
*Heloderma horridum* (Wiegmann, 1829)	3,4	LC	A	M (11)	4
**Family IGUANIDAE (1)**					
*Ctenosaura pectinata* (Wiegmann, 1834)	4	NE	NL	H (15)	1
**Family PHRYNOSOMATIDAE (30)**					
*Cophosaurus texanus* Troschel, 1852	1	LC	A	H (14)	2
*Holbrookia approximans* Baird, 1859	1,2	NE	NL	H (14)	1
*Holbrookia elegans* Bocourt, 1874	4	LC	NL	M (13)	2
*Phrynosoma cornutum* (Harlan, 1824)	1,2	LC	NL	M (11)	2
*Phrynosoma modestum* Girard, 1852	1	LC	NL	M (12)	2
*Phrynosoma orbiculare* (Linnaeus, 1758)	2,3	LC	A	M (12)	1
*Phrynosoma ornatissimum* (Girard, 1858)	2,3	NE	NL	NE	2
*Sceloporus albiventris* Smith, 1939	4	NE	NL	H (16)	1
*Sceloporus bimaculosus* Phelan & Brattstrom, 1955	1	NE	NL	NE	2
*Sceloporus bulleri* Boulenger, 1895	3	LC	NL	H (15)	1
*Sceloporus clarkii* Baird & Girard, 1852	4	LC	NL	M (10)	2
*Sceloporus cowlesi* Lowe & Norris, 1956	1	NE	NL	M (13)	2
*Sceloporus grammicus* Wiegmann, 1828	1,3	LC	Pr	L (9)	2
*Sceloporus heterolepis* Boulenger, 1895	3	LC	NL	H (14)	1
*Sceloporus jarrovii* Cope, 1875	1,3	LC	NL	M (11)	2
*Sceloporus lemosespinali* Lara-Góngora, 2004	3	DD	NL	H (16)	1
*Sceloporus maculosus* Smith, 1934	1	V	Pr	H (16)	1
*Sceloporus melanorhinus* Bocourt, 1876	3	LC	NL	L (9)	4
*Sceloporus merriami* Stejneger, 1904	1	LC	NL	M (13)	2
*Sceloporus nelsoni* Cochran, 1923	4	LC	NL	M (13)	1
*Sceloporus poinsettii* Baird & Girard, 1852	1,2,3	LC	NL	M (12)	2
*Sceloporus scalaris* Weigmann, 1828	2,3,4	LC	NL	M (12)	1
*Sceloporus shannonorum* Langebartel, 1959	3	NE	NL	H (15)	1
*Sceloporus slevini* Smith, 1937	3	LC	NL	M (11)	2
*Sceloporus spinosus* Weigmann, 1828	2	LC	NL	M (12)	1
*Sceloporus torquatus* Weigmann, 1828	1	LC	NL	M (11)	1
*Uma paraphygas* Williams, Chrapliwy & Smith, 1959	1	NT	P	H (17)	1
*Urosaurus bicarinatus* (Duméril, 1856)	4	LC	NL	M (12)	1
*Urosaurus ornatus* (Baird & Girard, 1852)	1	LC	NL	M (10)	2
*Uta stansburiana* Baird & Girard, 1852	1	LC	A	L (7)	2
**Family PHYLLODACTYLIDAE (1)**					
*Phyllodactylus tuberculosus* Wiegmann, 1834	4	LC	NL	L (8)	4
**Family SCINCIDAE (5)**					
*Plestiodon bilineatus* (Tanner, 1958)	3	NE	NL	M (13)	1
*Plestiodon callicephalus* (Bocourt, 1879)	4	LC	NL	M (12)	2
*Plestiodon lynxe* (Wiegmann, 1834)	3	LC	Pr	M (10)	1
*Plestiodon obsoletus* Baird & Girard, 1852	1	LC	NL	M (11)	2
*Scincella lateralis* (Say, 1823)	1	LC	Pr	M (13)	2
**Family TEIIDAE (4)**					
*Aspidoscelis costatus* (Cope, 1878)	4	NE	Pr	M (11)	1
*Aspidoscelis gularis* (Baird & Girard, 1852)	1	LC	NL	L (9)	2
*Aspidoscelis inornatus* (Baird, 1859)	1	LC	NL	H (14)	2
*Aspidoscelis marmoratus* (Baird & Girard, 1852)	1	NE	NL	H (14)	2
**Family XANTUSIDAE (2)**					
*Xantusia bolsonae* Webb, 1970	1	DD	P	H (17)	0
*Xantusia extorris* Webb, 1965	1	LC	NL	H (15)	1
**ORDER SQUAMATA**					
**SUBORDER SERPENTES (61)**					
**Family BOIDAE (1)**					
*Boa sigma* (Smith, 1943)	4	NE	NL	H (15)	1
**Family COLUBRIDAE (31)**					
*Arizona elegans* Kennicott, 1859	1,2	LC	NL	L (5)	2
*Bogertophis subocularis* (Brown, 1901)	1,2	LC	NL	H (14)	2
*Conopsis nasus* Günther, 1858	3	LC	NL	M (11)	1
*Drymarchon melanurus* (Duméril, Bibron & Duméril, 1854)	3,4	LC	NL	L (6)	3
*Gyalopion canum* (Cope, 1861)	1	LC	NL	L (9)	2
*Lampropeltis alterna* (Brown, 1901)	1,2,3	LC	A	H (14)	2
*Lampropeltis mexicana* (Garman, 1884)	3	LC	A	H (15)	1
*Lampropeltis splendida* (Baird & Girard, 1853)	1,2	NE	NL	M (12)	2
*Lampropeltis webbi* Bryson, Dixon & Lazcano, 2005	3	DD	NL	H (16)	1
*Leptophis diplotropis* (Günther, 1872)	4	LC	A	H (14)	1
*Masticophis bilineatus* Jan, 1863	4	LC	NL	M (11)	2
*Masticophis flagellum* (Shaw, 1802)	1,2,3	LC	A	L (8)	2
*Masticophis mentovarius* (Duméril, Bibron & Duméril, 1854)	2	LC	A	L (6)	4
*Masticophis taeniatus* (Hallowell, 1852)	1,2	LC	NL	M (10)	2
*Mastigodryas cliftoni* (Hardy, 1964)	3	NE	NL	H (14)	1
*Oxybelis aeneus* (Wagler, 1824)	4	NE	NL	L (5)	3
*Pantherophis emoryi* (Baird & Girard, 1853)	1	LC	NL	M (13)	2
*Pituophis catenifer* (Blainville, 1835)	1,2	LC	NL	L (9)	2
*Pituophis deppei* (Duméril, 1853)	2,3	LC	A	H (14)	1
*Pseudoficimia frontalis* (Cope, 1864)	4	LC	NL	M (13)	1
*Rhinocheilus lecontei* Baird & Girard, 1853	1	LC	NL	L (8)	2
*Salvadora bairdi* Jan, 1860	3	LC	Pr	H (15)	1
*Salvadora deserticola* Schmidt, 1940	1	NE	NL	H (14)	2
*Salvadora grahamiae* Baird & Girard, 1853	3	LC	NL	M (10)	2
*Senticolis triaspis* (Cope, 1866)	2,3	LC	NL	L (6)	3
*Sonora semiannulata* Baird & Girard, 1853	1	LC	NL	L (5)	2
*Tantilla atriceps* (Günther, 1895)	1	LC	A	M (11)	2
*Tantilla bocourti* (Günther, 1895)	3	LC	NL	L (9)	1
*Tantilla nigriceps* Kennicott, 1860	1	LC	NL	M (11)	2
*Tantilla wilcoxi* Stejneger, 1902	2,3	LC	NL	M (10)	2
*Trimorphodon tau* Cope, 1870	4	LC	NL	M (13)	1
**Family DIPSADIDAE (7)**					
*Diadophis punctatus* (Linnaeus, 1766)	1,3	LC	NL	L (4)	2
*Geophis dugesii* Bocourt, 1883	3	LC	NL	M (13)	1
*Heterodon kennerlyi* Kennicott, 1860	1,2	NE	NL	M (11)	2
*Hypsiglena jani* Dugès, 1865	1	NE	NL	L (6)	2
*Hypsiglena torquata* (Günther, 1860)	4	LC	Pr	L (8)	1
*Leptodeira splendida* Günther, 1895	4	LC	NL	H (14)	1
*Rhadinaea laureata* (Günther, 1868)	3	LC	NL	M (12)	1
**Family ELAPIDAE (1)**					
*Micrurus tener* Baird & Girard, 1853	1	LC	NL	M (11)	2
**Family LEPTOTYPHLOPIDAE (1)**					
*Rena segrega* (Klauber, 1939)	1	NE	NL	L (8)	2
**Family NATRICIDAE (12)**					
*Adelophis foxi* Rossman & Blaney, 1968	3	DD	Pr	H (16)	0
*Nerodia erythrogaster* (Forster, 1771)	1	LC	A	M (11)	2
*Storeria storerioides* (Cope, 1866)	3	LC	NL	M (11)	1
*Thamnophis cyrtopsis* (Kennicott, 1860)	1,2,3,4	LC	A	L (7)	3
*Thamnophis eques* (Reuss, 1834)	1,2,3	LC	A	L (8)	2
*Thamnophis errans* Smith, 1942	3	LC	NL	H (16)	1
*Thamnophis marcianus* (Baird & Girard, 1853)	1	LC	A	M (10)	3
*Thamnophis melanogaster* (Wiegmann, 1830)	1,2,3,4	E	A	H (15)	1
*Thamnophis nigronuchalis* Thompson, 1957	3	DD	Pr	M (12)	0
*Thamnophis pulchrilatus* (Cope, 1885)	3	LC	NL	H (15)	1
*Thamnophis unilabialis* Tanner, 1985	1,3	NE	NL	NE	1
*Thamnophis validus* (Kennicott, 1860)	2	LC	NL	M (12)	1
**Family TYPHLOPIDAE (1)**					
*Indotyphlops braminus* (Daudin, 1803)	1,2	N/A	N/A	N/A	IN
**Family VIPERIDAE (7)**					
*Crotalus atrox* Baird & Girard, 1853	1,2	LC	Pr	L (9)	2
*Crotalus lepidus* (Kennicott, 1861)	1,2,3	LC	Pr	M (12)	2
*Crotalus molossus* Baird & Girard, 1853	1,2,3	LC	Pr	L (8)	2
*Crotalus pricei* Van Denburgh, 1895	3	LC	Pr	H (14)	2
*Crotalus scutulatus* (Kennicott, 1861)	1,2	LC	Pr	M (11)	2
*Crotalus stejnegeri* Dunn, 1919	3,4	V	A	H (17)	1
*Crotalus willardi* Meek, 1905	2,3	LC	Pr	M (13)	2
**ORDER TESTUDINES (5)**					
**Family EMYDIDAE (1)**					
*Trachemys gaigeae* (Hartweg, 1939)	1	V	NL	H (18)	2
**Family KINOSTERNIDAE (3)**					
*Kinosternon durangoense* Iverson, 1979	1	DD	NL	H (16)	1
*Kinosternon hirtipes* (Wagler, 1830)	1,2,3,4	LC	Pr	M (10)	2
*Kinosternon integrum* LeConte, 1854	2,3	LC	Pr	M (11)	1
**Family TESTUDINIDAE (1)**					
*Gopherus flavomarginatus* Legler, 1959	1	V	P	H (19)	1

**Table 2.** Summary of native species present in Durango by Family, Order or Suborder, and Class. Status summary indicates the number of species found in each IUCN conservation status in the order DD, LC, V, NT, E, CE (see Table 1 for abbreviations; in some cases species have not been assigned a status by the IUCN and therefore these may not add up to the total number of species in a taxon). Mean EVS is the mean Environmental Vulnerability Score, scores ≥ 14 are considered high vulnerability (Wilson et al. 2013a,b) and conservation status in Mexico according to SEMARNAT (2010) in the order NL, Pr, A, P (see Table 1 for abbreviations).

Taxon	Genera	Species	IUCN	EVS	SEMARNAT
Class Amphibia					
Order Anura	13	32	2,24,2,1,1,0	10.2	23,8,1,0
Bufonidae	3	11	0,8,0,1,0,0	9.9	10,1,0,0
Craugastoridae	1	4	1,2,1,0,0,0	12.8	3,1,0,0
Eleutherodactylidae	1	3	1,1,0,0,1,0	15.3	2,1,0,0
Hylidae	4	6	0,6,0,0,0,0	8.5	5,1,0,0
Microhylidae	1	1	0,1,0,0,0,0	9	0,1,0,0
Ranidae	1	5	0,4,1,0,0,0	10.4	1,3,1,0
Scaphiopodidae	2	2	0,2,0,0,0,0	4.5	2,0,0,0
Order Caudata	1	3	1,2,0,0,0,0	12.7	1,2,0,0
Ambystomatidae	1	3	1,2,0,0,0,0	12.7	1,2,0,0
SUBTOTAL	14	35	3,26,2,1,1,0	10.4	24,10,1,0
Class Reptilia					
Order Squamata					
Suborder Lacertilia	21	53	2,38,1,1,0,0	12.5	37,9,5,2
Anguidae	3	4	0,3,0,0,0,0	11	2,2,0,0
Crotaphytidae	2	2	0,2,0,0,0,0	13	0,1,1,0
Dactyloidae	1	1	0,1,0,0,0,0	13	1,0,0,0
Eublepharidae	1	2	0,2,0,0,0,0	15.5	1,1,0,0
Helodermatidae	1	1	0,1,0,0,0,0	11	0,0,1,0
Iguanidae	1	1	0,0,0,0,0,0	15	1,0,0,0
Phrynosomatidae	7	30	1,21,1,1,0,0	12.5	24,2,3,1
Phyllodactylidae	1	1	0,1,0,0,0,0	8	1,0,0,0
Scincidae	2	5	0,4,0,0,0,0	11.8	3,2,0,0
Teiidae	1	4	0,2,0,0,0,0	12	3,1,0,0
Xantusidae	1	2	1,1,0,0,0,0	16	1,0,0,1
Suborder Serpentes	33	60	3,46,1,0,1,0	11	39,10,11,0
Boidae	1	1	0,0,0,0,0,0	15	1,0,0,0
Colubridae	19	31	1,26,0,0,0,0	10.7	25,1,5,0
Dipsidae	6	7	0,5,0,0,0,0	9.7	6,1,0,0
Elapidae	1	1	0,1,0,0,0,0	11	1,0,0,0
Leptotyphlopidae	1	1	0,0,0,0,0,0	8	1,0,0,0
Natricidae	4	12	2,8,0,0,1,0	12.1	5,2,5.0
Viperidae	1	7	0,6,1,0,0,0	12	0,6,1,0
Order Testudines	3	5	1,2,2,0,0,0	14.8	2,2,0,1
Emydidae	1	1	0,0,1,0,0,0	18	1,0,0,0
Kinosternidae	1	3	1,2,0,0,0,0	12.3	1,2,0,0
Testudinidae	1	1	0,0,1,0,0,0	19	0,0,0,1
SUBTOTAL	57	118	6,86,4,1,1,0	11.8	78,21,16,3
TOTAL	68	153	9,112,6,2,2,0	11.5	102,31,17,3

### General distribution

Twenty-one of the 36 species of Amphibians that inhabit Durango are endemic to Mexico, 13 of them are limited to the Sierra Madre Occidental or to the Pacific Coast and the lowlands of the Sierra Madre Occidental (Table 1). Three more are species typical of the Mexican Plateau (Table 1). Another five have wide distributions that include parts of both Sierras Madre (Occidental and Oriental) and part of the Mexican Plateau (Table 1).

Of the 15 amphibian species of Durango that are not endemic to Mexico, one is an introduced species (*Rana catesbeiana*), and eleven more are found in the USA and Mexico (Table 1). The remaining three species have a wide distribution from southern USA to Central or South America (Table 1).

Twenty-four of the 54 species of lizards that occur in the state are endemic to Mexico, one of them to the state of Durango (*Xantusia bolsonae*), three more have narrow distributions in northeastern Durango: *Sceloporus maculosus* limited to the Río Nazas drainage in Durango and Coahuila; *Uma paraphygas* limited to the Bolsón de Mapimí of southeastern Chihuahua, southwestern Coahuila, and northeastern Durango; and *Xantusia extorris* limited to northeastern Durango and adjacent Coahuila. Two more are restricted to small areas in the Sierra Madre Occidental: *Sceloporus lemosespinali* to eastern Sonora, northern Chihuahua, and extreme northwestern Durango; and *S. shannonorum* in central Durango to extreme northern Jalisco. Another ten species that occur in Durango and are endemic to Mexico are typical to the Pacific Coast and/or the Sierra Madre Occidental: *Anolis nebulosus*, *Coleonyx fasciatus*, *Ctenosaura pectinata*, *Sceloporus albiventris*, *S. bulleri*, *S. heterolepis*, *S. nelsoni*, *Urosaurus bicarinatus*, *Plestiodon bilineatus*, and *Aspidoscelis costatus*. One more is a species typical of the Chihuahuan Desert: *Holbrookia approximans*. Another species is typical of the Sierra Madre Oriental, with an isolated population occurring in southern Durango: *Plestiodon lynxe*. One more occurs in southern Mexico in the state of Puebla, Hidalgo, Oaxaca, and Chiapas, with isolated populations in Aguascalientes, Jalisco, and southwestern Durango: *Gerrhonotus liocephalus*. The remaining five lizard species endemic to Mexico have a wide distribution occurring in both Sierras Madres (Occidental and Oriental): *Barisia ciliaris*, and even in the Transvolcanic Belt of central Mexico (*Phrynosoma orbiculare*), or are species typical of the Mexican Plateau: *Sceloporus scalaris*, *S. spinosus*, and *S. torquatus*.

The remaining 30 species of lizards that inhabit Durango are not endemic to Mexico. Twenty-six of the non-endemics are species found in the USA and Mexico (Table 1). Three are found from northern Mexico to Central America (Table 1). The last one, *Hemidactylus turcicus*, is introduced to Durango.

Twenty-four of the 61 species of snakes that occur in Durango are endemic to Mexico. Two of them to Durango: *Adelophis foxi* and *Thamnophis nigronuchalis*. Four others have a narrow distribution in the Sierra Madre Occidental: *Lampropeltis webbi* (Pacific slope of the Sierra Madre Occidental near the Durango – Sinaloa border); *Thamnophis errans* (from central Chihuahua, Durango and adjacent Zacatecas); *Thamnophis unilabialis* (eastern Sonora and western Chihuahua to northern Durango); and *Crotalus stejnegeri* (western Durango and adjacent southern Sinaloa). Eight more are typical species of the Pacific slopes of the Sierra Madre Occidental: *Boa sigma, Leptophis diplotropis*, *Mastigodryas cliftoni*, *Pseudoficimia frontalis*, *Geophis dugesii*, *Hypsiglena torquata*, *Leptodeira splendida*, and *Thamnophis validus*. Another nine of the endemic snakes have a wide distribution in central Mexico that include the Mexican Plateau and/or the Transvolcanic Belt of central Mexico and the Sierra Madre Occidental and in some cases even the Sierra Madre Oriental: *Conopsis nasus*, *Lampropeltis mexicana*, *Pituophis deppei*, *Salvadora bairdi*, *Tantilla bocourti*, *Trimorphodon tau*, *Rhadinaea laureata*, *Storeria storerioides*, and *Thamnophis melanogaster*. The remaining endemic species, *Thamnophis pulchrilatus*, has a spotty distribution in highlands of the Sierra Madre Occidental and the Sierra Madre Oriental.

Thirty snake species that are found in Durango are distributed from the USA to Mexico (Table 1). Five more species are found from central or southern USA to Central or South America (Table 1). One species ranges from Mexico to northeastern South America: *Masticophis mentovarius*. The last species that inhabits Durango and is not endemic to Mexico is an introduced species to Mexico, *Indotyphlops braminus*.

Three of the five species of turtles that inhabit Durango are endemic to Mexico, two to the Bolsón de Mapimí in southeastern Chihuahua, southwestern Coahuila, and northeastern Durango: *Kinosternon durangoense* and *Gopherus flavomarginatus*. The other is widely distributed in the lowlands of western Mexico and throughout the central and southern portion of the Mexican Plateau: *Kinosternon integrum* (it is not native to the Valley of Mexico but has been introduced there). The two non-endemic species of turtles are found from southwestern USA to northern Mexico: *Trachemys gaigeae* and *Kinosternon hirtipes*.

### Habitat types

The Sierra habitat type (46.1%) and the arid-semiarid habitat type (42.8%) had the highest percentages of the herpetofauna in Durango, whereas both the valley (29.9%) and Quebradas (24.0%) habitat types had a lower percentage (Table 3). For amphibians alone, the Sierra habitat type had slightly more than 50% of the species in Durango (52.8%) followed by the valley habitat type (41.7%) and Quebradas habitat type (30.6%). As might be expected, the arid-semi-arid habitat type had the fewest amphibian species (19.4%; Table 3). This distribution of species is also found when examining anuran species (Table 3). For salamanders, species are almost exclusively found in the Sierra habitat type, with one species found in the valley habitat type, and none in the arid-semi-arid and Quebradas habitat types (Table 3). Reptiles showed a different pattern, with the most species being found in the arid-semiarid habitat type (50%) and the Sierra habitat type (44.1%), with the valleys (26.3%) and the Quebradas (22.0%) having fewer species. This pattern is found in both lizards and snakes (Table 3), and is primarily driven by the most diverse families in these groups (e.g., Phrynosomatidae, Colubridae, and Natricidae). Turtles are found in the four habitat types with 80% of the species occurring in the arid-semiarid habitat type and less than half of the species found in the other three habitat types.

**Table 3.** Summary of the number of native species (% of total number of species of taxonomic group in Durango in parentheses) in different taxonomic groups found in different habitat types in Durango, Mexico (see text for description of the habitat types).

Taxon	Arid-Semiarid	Valleys	Sierras	Quebradas
Amphibia	7 (19.4)	15 (41.7)	19 (52.8)	11 (30.6)
Caudata	0 (0)	1 (33.3)	3 (100)	0 (0)
Anura	7 (21.2)	14 (42.4)	16 (48.5)	11 (33.3)
Reptilia	59 (50)	31 (26.3)	52 (44.1)	26 (22.0)
Testudines	4 (80)	2 (40)	2 (40)	1 (20)
Squamata	55 (48.7)	29 (25.7)	50 (44.2)	25 (22.1)
Lacertilia	26 (49.0)	8 (15.1)	20 (37.7)	13 (24.5)
Serpentes	29 (48.3)	21 (35)	30 (50)	12 (20)
Total	66 (42.8)	46 (29.9)	71 (46.1)	37 (24.0)

### Comparisons with neighboring states

**Figure 5.**
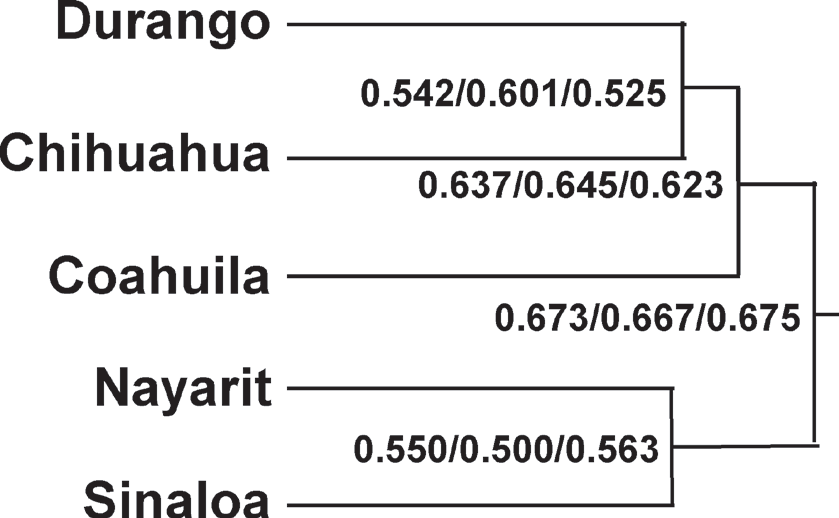
Results of cluster analysis of the herpetofaunas of Durango and its neighboring states (Chihuahua, Coahuila, Nayarit, and Sinaloa). The distances provided are Euclidean distances for the entire herpetofauna, reptiles only, and amphibians only, respectively.

Overall, Durango shares the most species with Chihuahua (Table 4). This holds true for amphibians; however, Durango shares almost as many species of amphibians with Sinaloa and Nayarit as with Chihuahua. For reptiles, Durango and Chihuahua share the most species by a large margin over the other states (Table 4). The cluster analysis recovered the same tree structure for Durango and its neighboring states when the entire herpetofauna, reptiles, and amphibians are each considered (Figure 5). In each case, Durango and Chihuahua made a cluster and Nayarit and Sinaloa made another cluster. In addition, Coahuila formed a cluster with the Durango-Chihuahua pairing. Such a pattern likely reflects the fact that Durango, Chihuahua, and Coahuila all have extensive tracts of Chihuahuan Desert habitats. Similarities and differences in species among Durango and its neighboring states likely is the result of the habitats and vegetation types found in each state (see also Smith and Lemos-Espinal 2015, Lemos-Espinal and Smith 2016, Lemos-Espinal et al. 2017). Such results suggest that the conservation of the herpetofauna of this region will need habitat specific conservation plans that cross state borders and will require integration of state, regional, and country-level efforts.

**Table 4.** Summary of the numbers of species shared between Durango and neighboring Mexican states (not including introduced species). The percent of species from Durango shared by a neighboring state are given in parentheses. – indicates either Durango or the neighboring state has no species in the taxonomic group, thus no value for shared species is provided.

Taxon	Durango	Chihuahua	Sinaloa	Nayarit	Coahuila
Class Amphibia	35	23 (65.7)	20 (57.1)	19 (54.2)	11 (30.6)
Order Caudata	3	2 (66.7)	1 (33.3)	1 (33.3)	0 (0)
Ambystomatidae	3	2 (66.7)	1 (33.3)	1 (33.3)	0 (0)
Order Anura	32	21 (65.6)	19 (59.4)	18 (56.2)	11 (33.3)
Bufonidae	11	8 (72.7)	7 (63.6)	4 (36.4)	4 (36.4)
Craugastoridae	4	2 (50)	3 (75)	3 (75)	1 (25)
Eleutherodactylidae	3	0 (0)	2 (66.7)	2 (66.7)	0 (0)
Hylidae	6	4 (66.7)	4 (66.7)	5 (83.3)	2 (33.3)
Microhylidae	1	1 (100)	0 (0)	0 (0)	1 (100)
Ranidae	5	4 (80)	2 (40)	2 (40)	1 (16.7)
Scaphiopodidae	2	2 (100)	1 (50)	2 (100)	2 (100)
Class Reptilia	118	90 (76.3)	53 (44.9)	55 (46.6)	63 (53.4)
Order Testudines	5	5 (100)	1 (20)	2 (40)	4 (80)
Emydidae	1	1 (100)	0 (0)	0 (0)	1 (100)
Kinosternidae	3	3 (100)	1 (33.3)	2 (66.7)	2 (66.7)
Testudinae	1	1 (100)	0 (0)	–	1 (100)
Order Squamata	113	85 (75.2)	52 (46.0)	53 (46.9)	59 (52.2)
Suborder Lacertilia	53	37 (69.8)	20 (37.7)	22 (41.5)	25 (47.2)
Anguidae	4	3 (75)	2 (50)	2 (50)	1 (25)
Crotaphytidae	2	2 (100)	–	–	2 (100)
Dactyloidae	1	1 (100)	1 (100)	1 (100)	0 (0)
Eublepharidae	2	1 (50)	1 (50)	0 (0)	1 (50)
Helodermatidae	1	1 (100)	1 (100)	1 (100)	0 (0)
Iguanidae	1	0 (0)	1 (100)	1 (100)	–
Phrynosomatidae	30	21 (70)	11 (36.7)	13 (43.3)	15 (50)
Phyllodactylidae	1	1 (100)	1 (100)	1 (100)	–
Scincidae	5	3 (60)	1 (20)	2 (40)	2 (40)
Teiidae	4	4 (100)	1 (25)	1 (25)	3 (75)
Xantusidae	2	–	–	–	1 (50)
Suborder Serpentes	60	48 (80)	32 (53.3)	31 (51.7)	34 (56.7)
Boidae	1	1 (100)	1 (100)	1 (100)	0 (0)
Colubridae	31	25 (80.6)	20 (64.5)	15 (48.4)	18 (58.1)
Dipsidadae	7	6 (85.7)	4 (57.1)	4 (57.1)	3 (42.8)
Elapidae	1	0 (0)	0 (0)	0 (0)	1 (100)
Leptotyphlopidae	1	1 (100)	0 (33.3)	0 (0)	1 (100)
Natricidae	12	9 (75)	3 (25)	8 (66.7)	6 (50)
Viperidae	7	6 (85.7)	4 (57.1)	3 (42.8)	5 (71.4)
TOTAL	153	113 (73.8)	73 (47.7)	74 (48.4)	74 (48.0)

### Conservation status

Overall, 7.6% of the amphibian and reptile species were IUCN listed (i.e., Vulnerable, Near Threatened, Endangered, or Critically Endangered), but 33.3% were placed in a protected category by SEMARNAT and 29.3% categorized at high risk by the EVS (Tables 1, 2). For amphibians, 12.1% were IUCN listed, 31.4% protected by SEMARNAT, and 20% at high risk according to the EVS (Tables 1, 2). For reptiles, 6.1% were listed in IUCN, 33.9% protected by SEMARNAT, and 32.2% at high risk by EVS. These results suggest that the herpetofauna of Durango is considered to be of relatively low conservation concern at a global scale, but at a national level, there is much greater conservation concern. There are several taxa that, based on their IUCN listing, SEMARNAT category, or their EVS, are of conservation concern. Families that include species of particular conservation concern include Eleutherodactylidae, Eublepharidae, Iguanidae, Phrynosomatidae, Xantusidae, Boidae, Colubridae, Natricidae, Emydidae, and Testudinidae (Tables 1, 2). The IUCN, SEMARNAT, and EVS categories are based on global or country-level assessments, and it is likely that there are amphibians and reptiles whose conservation status in Durango is not accurately assessed by the global level assessment. Additional assessments at the state level in Durango, and other Mexican states, are needed to establish conservation or management needs for states, or even regions.

To help determine which habitat types within Durango may house species of particular conservation concern, the conservation statuses of reptile and amphibian taxa in each habitat type found in Durango was summarized. None of the amphibians in the arid-semiarid habitats and Quebradas were in protected IUCN categories (VU, NT, EN, CE), 7.1% in the valleys, and 25% in the Sierra habitat. For SEMARNAT categories, 42.8% of amphibians in the arid-semiarid habitats, 26.7% in the valleys, 33.3% in the Sierra habitat, and 18.2% in the Quebradas were listed. For EVS, 100% of the amphibians in the arid-semiarid habitat were in the low category. Almost half (46.6%) of the amphibians in the valley habitat were in the low category, 40% in the medium category, and 13.3% in the high category. In the Sierra habitat type, 26.3% of amphibians were in the low category, 42.1% in the medium, and 31.6% in the high. For the Quebradas habitat, 45.4% were in the low and medium categories and 9.1% in the high. Based on this summary, it is clear that for amphibians, the Sierra habitat has the most at risk species and the arid-semiarid habitat has relatively fewer at risk species. For amphibians, therefore, the Sierra habitat would appear to be a priority target for conservation efforts.

For the IUCN listings, all habitat types had relatively few species of reptiles in the protected categories (arid-semiarid, 8.5%; valleys, 3.2%; Sierra, 3.8%; and Quebradas, 7.7%). However, 39% of reptiles in the arid-semiarid habitat, 41.9% from the valley habitat, 42.3% from the Sierra habitat, and 30.8% from the Quebradas habitat were in the protected SEMARNAT categories. For the arid-semiarid habitat type, 28.1% of reptiles were in the low EVS category, 43.8% in the medium, and 28.1% in the high. In valleys, 29% of the reptiles were in the low, 51.6% in the medium, and 19.4% in the high. Of the reptiles in the Sierra habitat type, 21.6% were in the low, 45.1% in the medium, and 33.3% in the high categories. For the Quebradas habitat type, 19.2% were in the low EVS category, 50% in the medium, and 30.8% in the high. In contrast to amphibians, at risk reptile species are more evenly distributed across the habitat types. Therefore, conservation efforts for reptiles should address all habitat types.

Three non-native species of amphibians and reptiles were documented in Durango: *R. catesbeiana*, *H. turcicus*, and *I. braminus*. Non-native species can negatively affect native herpetofaunal communities in Mexico (see Wilson and Townsend 2010). Of the three non-native species, *R. catesbeiana* is of particular concern. *Rana catesbeiana* is known to have many impacts on native communities as a competitor, predator, and disease vector on a global scale (reviewed in Moutou and Pastoret 2010; Kraus 2015), as well as in Mexico (e.g., Luja and Rodriguez-Estrella 2010; Becerra Lopez et al. 2017). The potential impacts of *H. turcicus* are less well documented, but its congener *H. frenatus* has affected native herpetofauna through competition (reviewed in Punzo 2005; Kraus 2015). The impacts of *I. braminus* are, to our knowledge, unstudied, even though it has been widely introduced around the world (see Borroto-Páez et al. 2015). There is thus the potential for these non-native amphibians and reptiles to have negative impacts on the native herpetofauna, and other organisms, of Durango. The extent of these potential impacts need to be evaluated further.

Hopefully, this list of amphibian and reptile species in Durango with their global and country-level conservation statuses will prompt further investigations into the herpetofauna of this state, which could provide the needed information to allow for state- or regional-specific, or even habitat type, conservation measures to be undertaken.
